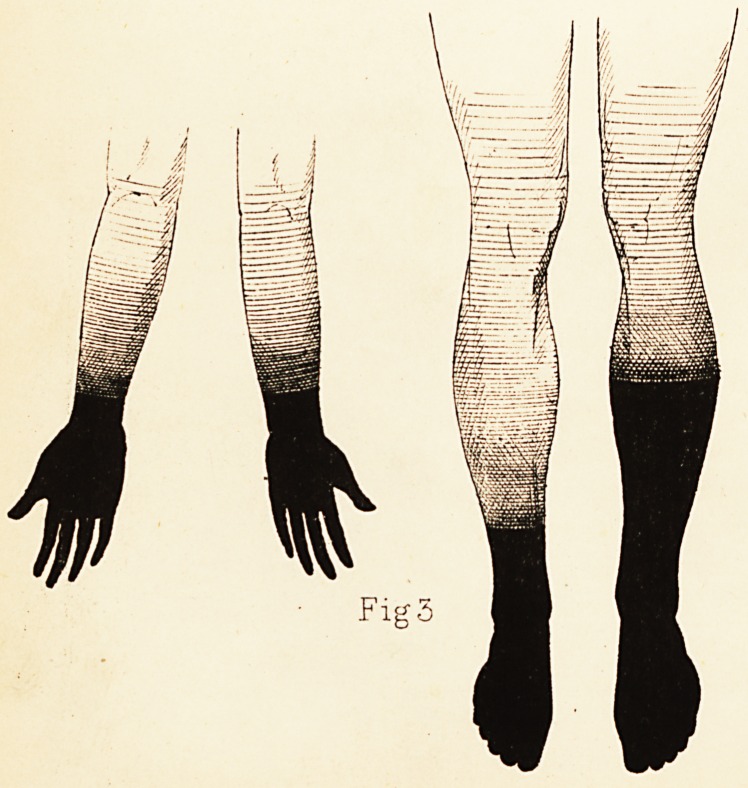# Peripheral Neuritis

**Published:** 1887-06

**Authors:** J. Michell Clarke

**Affiliations:** Assistant Physician to the Bristol General Hospital, and Demonstrator of Physiology at the Bristol Medical School


THE BRISTOL

### Medico-Chirurgical Journal.

JUNE, 1887.

### PERIPHERAL NEURITIS.

Read before the Bristol Medico-Chirurgical Society

BY

J. MICHELL CLARKE, M.A., M.B. CANTAB.,

*Assistant Physician to the Bristol General Hospital, and Demonstrator of Physiology at the Bristol Medical School.*

By the term Peripheral or Multiple Neuritis is meant an inflammation of the nerve-trunks after their exit from the cranial and intervertebral foramina. I propose to confine myself to those cases only in which many nerve-trunks are invaded progressively, and to the varieties of this large group of diseases of which I have here some instances. Excluding, then, altogether cases of neuritis limited to single nerves, such as optic and facial neuritis, the diseases grouped together under the title of peripheral or multiple neuritis arise from many different causes; and it is possible that many of them will be found to be essentially distinct affections. It is only quite lately that these diseases have been separated from the longer known affections of the cerebrospinal system, with which they were formerly confounded; although one variety, "alcoholic neuritis," was described by Lettsom in 1789, under the title of "History of Some of the Effects of Hard Drinking;" while one of Graves's clinical lectures deals with the diseases of the nervous system arising in the extremities and spreading towards the centre, and in it is described a remarkable epidemic witnessed by him in Paris in 1840, and evidently an epidemic of peripheral neuritis. Still, it has not been generally recognised until recently that there are diseases of the nervous system which begin in the periphery, with successive invasion of many nerve-trunks, and spread towards the centre; and the paralyses due to diphtheria and lead-poisoning respectively were the first to be recognised as of this nature, and to suggest the existence of cases presenting the same or similar symptoms, but due to other causes.

As to the pathology of the affection, two kinds of neuritis are known—a parenchymatous and an interstitial: the former probably simply degenerative, consisting of disintegration and absorption of the myelin of the medullary sheath, and breaking up of the axis-cylinder; the latter (interstitial) consisting of inflammatory changes in the endo- and peri-neurium, with formation of much new connective tissue pressing upon and so destroying the true nerve-substance. Gambault, by inducing chronic lead-poisoning in pigs, produced a parenchymatous neuritis in which a segment lying between two nodes of Ranvier is diseased, while the segments on the other side of these nodes remain healthy; and in two cases of alcoholic paralysis this condition was found to exist by Drs. Dreschfeld and Grainger Stewart.

Hadden, Eichhorst, and other observers have found the change to be an interstitial neuritis. There was great increase of connective tissue around and between the bundles of nerve-fibrils, which were deficient in number, and more or less disorganised, apparently from pressure.

Ross states that Gambault has found in alcoholic paralysis proliferation of the nuclei of the endoneurium and sheath of Schwann at a certain level of the diseased nerve, while the medullary sheaths and axis-cylinders were simply attenuated, probably from compression. Below this level the changes were those of the ordinary Wallerian degeneration. It is, therefore, possible that at a certain level of the nerve a perineuritis may be observed cutting off the fibres below from their trophic centres, so that the latter undergo the usual descending degeneration.

*Varieties.*—Peripheral neuritis may be due (1) to the mineral poisons of lead, arsenic, and copper; (2) to alcohol and the fumes of bisulphide of carbon—a neuritis of this variety occurs in some cases of diabetes; (3) to animal poisons of syphilis, diphtheria, typhoid, typhus, and the other specific fevers. Ross has recently reported a case occurring after rheumatic fever, and Dr. Chapin, of New York, one or two cases of apparently malarial origin. The disease known in Japan as *kakké* or *beri-beri* seems to be a variety of multiple neuritis, and the lesions of anæsthetic leprosy are due to it. Finally, there are several cases reported of so-called idiopathic neuritis, perhaps arising from exposure to cold. The disease seems to affect tubercular subjects more than others.

*Symptoms.*—Cases of acute or subacute multiple neuritis are often preceded by sensations of pins and needles, tingling or numbness in the toes and fingers. Dr. Allen Starr, of New York, thinks that the disease may stop here, and that the sensations so commonly complained of by women at the climacteric, of tingling with slight loss of power in the fingers, are due to neuritis. Vague rheumatic pains or cramps may occur. The onset is generally insidious, but there is occasionally slight fever. The numbness and deadness spread gradually up the extremities, and in a few days the patient may find that he has lost all power in his feet and hands. In all forms of peripheral neuritis, except that due to lead-poisoning, there are generally lancinating pains through the limbs, and sensations of burning, sometimes of great severity, and worse at night. As in other diseases in which the exciting cause is a poison circulating in the blood, the neuritis tends to involve both sides of the body symmetrically, though there are often considerable differences in the gravity of the symptoms on the two sides. In severe cases the paralysis may spread from the muscles of the extremities to those of the trunk, and even of the face and eyes; and death may occur from implication of the vagus, causing interference with respiration and swallowing. But in the great majority of cases the paralysis does not spread beyond the limbs; sooner or later the climax is reached, and recovery begins. This recovery may be so rapid as to make it seem doubtful whether any organic lesion has occurred, or so slow that the probability of complete restoration of function seems small. The paralysis is always of the atrophic variety—the muscles emaciate and soften, rapidly waste, and become flaccid. The cutaneous reflexes are absent in the limbs involved, the cremasteric and abdominal appear to be often exaggerated. The deep reflexes give valuable information as to the nature of the paralysis, especially the knee-jerk. This has been found to be absent in all the recent cases reported of alcoholic, diphtheritic, and other forms of peripheral neuritis affecting the legs. In one case reported by Oppenheimer, in which recovery took place in three months, the knee-jerk was found exaggerated at the onset before it finally disappeared; and in other cases, notably one of multiple neuritis from lead, the patellar tendon was found to be exaggerated by Ross, who states, that though absence of the knee-jerk is a valuable sign of peripheral neuritis, it is not absolutely conclusive, and must be taken together with the other symptoms. Now, though for the proper performance of the knee-jerk it is necessary that the reflex arc be intact, it has been almost certainly shown that the knee-jerk is not a reflex action, but due to sudden tension of the muscle exciting the end-plates of the muscular nerves.* Since peripheral neuritis begins at the endings of the nerves, before there is actual paralysis of these, I think that we should expect an antecedent stage of abnormal irritability; if the patient were then examined the knee-jerk would be found exaggerated, but afterwards would disappear, as in the observation of Oppenheimer quoted above. As I have had three cases of diphtheria under my care lately, I took the opportunity to investigate this point. In the first, the knee-jerk was decidedly exaggerated about ten days after the onset of the disease, when the patient complained of tingling, &c., in the feet; it then gradually became weaker and weaker, until it finally disappeared about ten days later, when there was well-marked paralysis of the feet and legs. In the second, the knee-jerk was exaggerated twelve days before its final disappearance with the onset of paralysis, and it disappeared in the same gradual manner. It is important to note that the front-tap contraction—a very delicate test of abnormal irritability of the calf muscles—

* Weir Mitchell, and Lewis of New York, and Lombard, *Int. Journ. Med. Sciences*; Gowers' *Diseases of Nervous System*, &c.

was present in both at the same time as the exaggerated knee-jerk. The cutaneous reflexes were present, but the patients were children, in whom they are always more readily obtained than in adults; so that one could not say they were increased. In the second case, I thought I detected ankle-clonus, slight and of brief duration, but still well marked, on one day at the same period—*i.e.* about ten days from the beginning of the disease, and twelve from complete disappearance of knee-jerk. Later, the front-tap contraction and plantar reflex disappeared with the knee-jerk. In the third case, the knee-jerk appeared to be somewhat exaggerated throughout, and there was no paralysis or subsequent disappearance of it. These cases then, so far as they go, are in favour of the view advanced above; and since most cases of multiple neuritis come under notice when the paralysis is well marked, the absence of knee-jerk will be almost invariably observed.*

The electrical reactions give conclusive evidence of the nature of the disease in most cases. Faradaic irritability is either greatly reduced or entirely lost, while the muscles react forcibly to a weak constant current; in other words, the R. D. is present. In most cases Faradaic irritability is simply lowered, not abolished, while the Galvanic test gives the degenerative reaction; but it is necessary to be aware that in some cases the irritability to both Faradaic and constant currents is simply lowered.†

* Since the above was written, Drs. Ad. Strümpell and Möbius have reported two cases of peripheral neuritis in which there was exaggeration of tendon reflexes, this exaggeration gradually disappearing as the patients improved. They had previously observed that the tendon reflexes of the arms were increased in a case of lead-palsy, and attribute the phenomenon to increased excitability of the afferent muscle-nerves

† Ross, *British Medical Journal*, January 1st, 1887.

Though the distribution of the paralysis varies according to the cause of the disease, predominance of paralysis of extensors is a diagnostic point. This extensor paralysis gives rise to wrist- and ankle-drop. The consequence of ankle-drop is approximation of the origins and insertions of the calf muscles, so that the foot is maintained in a position of talipes equinus or equino-varus; and in a case of alcoholic paralysis of Dr. Buzzard's the muscles of the calf became so contracted that the patient could not bring the heel to the ground, though he could still walk with difficulty. If the patient sits with his knees bent at a right angle and the soles of his feet resting flat on the floor, paralysis of extensors of ankle and toes will at once be detected by his inability to bend the foot up to an angle with the floor without moving the heel. In the arm, from the same excess of paralysis of extensors and shortening of distances between the insertions of flexors, the forearm is bent at an acute angle with the arm, the flexion of the wrist still further increased, and the fingers bent in towards the palm, from the unopposed action of flexors; a condition which it is necessary to distinguish from active spasm of the flexor muscles. Into the various deformities produced in this way I cannot enter. There is often hyperæsthesia of the skin in the early stages, which generally gives place later on to more or less anæsthesia, with retardation of sensory conduction and loss of tactile sensation. Tenderness and apparent enlargement of nerve-trunks affected may be made out. It is important to observe that in some cases there is no pain throughout, and that affection of sensation may be absent or so slightly marked as to be with difficulty elicited.

Vaso-motor disturbances generally in the form of hyperidrosis and trophic troubles in the shape of glossy skin, bullous and herpetic eruptions, are sometimes present; local asphyxia and even gangrene of the limbs have been noticed (Lancereaux and Myrtle, quoted by Ross). Bed-sores have been reported in two cases only; incontinence of urine and fæces are practically unknown, and ophthalmoplegia interna and externa absent except in diphtheritic cases.

The forms of neuritis due to lead-poisoning and to diphtheria are too well known to require description. The following case, however, illustrates how very slight the degree of throat affection may be in diphtheria, and yet be followed by paralysis: moreover, in this case the paralysis, after affecting the palate and larynx, passed to the legs, at an interval of over three months, and did not affect the eyes or any other muscles; in the legs the paralysis exhibited the typical symptoms of a mild peripheral neuritis, affecting both feet:

The patient was a young man, æt. 22, of robust appearance, who had always been healthy and temperate, and had never had syphilis. He complained of numbness and tingling in toes and feet, and loss of power in feet and legs, felt especially in walking. Close questioning brought out that he had had "quinsy" nearly four months ago, was not confined to bed by it, and—in answer to a leading question—that shortly afterwards he lost his voice, and that fluids ran out of his nose when drinking for two or three days afterwards. He was then quite well until three days ago, when present illness began. He walked with the peculiar gait described below, closely watched his feet when walking, and at once stumbled if he tried to walk with his eyes shut, but did not feel giddy.*

* In the figures the degree of anæsthesia, as tested by wire-brush and interrupted current of varying strength, is indicated by the degree of shading: absolute blackness meaning complete loss of sensation.

Plate 1.

Fig I

Fig 2

Fig 3

There was great loss of sensation over the feet and ankles, becoming complete over the toes. Loss of sensation to pain was more marked than the loss to touch. Fig. I. shows the extent of the anæsthesia. There was double ankle-drop, not quite so complete on left side as on right. Placed sitting in a chair with the feet flat on the floor, he was unable to elevate the toes from the ground. The big toe on each side curved down towards the sole. The knee-jerks and plantar reflexes were absent. Cremasteric and abdominal well marked.

R. D. obtained—no reaction at all to interrupted Faradaic current—for common extensor of toes and proper extensor of big toe on each side. There was diminished reaction to interrupted, increased to constant current for the other muscles of the leg. The thigh muscles reacted normally. Complete recovery took place in a little over six weeks.

Alcoholic neuritis occurs more often in women than men; it seems to be especially produced by drinking inferior spirits and liqueurs. The lower extremities are generally first paralysed; the paralysis begins with a double ankle-drop, and the extensor of the big toe is involved as frequently as the common extensor and peronei. This gives a curving down of the big toe into the sole, which was noted in the preceding case of diphtheritic paralysis, and is a characteristic of the ankle-drop of peripheral neuritis, contrasting markedly with the hyper-extension of the big toe in spastic paraplegia. The paralysis in these cases often remains limited to the legs, but in many the extensors of the forearm are affected. The muscles next involved in order are the quadriceps extensor of the thigh, triceps, muscles of calf, flexors of forearm, small muscles of hand and foot. If the paralysis extend further, the muscles of the back become so weak that the patient is unable to sit up in bed, and the other muscles of the limbs are affected. One of the chief features of this variety is the extreme tenderness of the muscles, so that the patient may scream if they are handled, and sometimes cannot bear the weight of the bedclothes. In later stages this hyperæsthesia is replaced by anæsthesia.*

The following case is one of alcoholic neuritis, presenting unusual features. The patient was a washerwoman, æt. 28, a big but rather flabby-looking woman. She had never had syphilis, but had been very intemperate, habitually drinking a large amount of beer and spirits. She complained of complete loss of power in right arm and partial loss in left, of dull aching pains in the right arm, worse at night. The loss of strength and co-ordination was especially marked in the wrist and hand, there was numbness in the hand and fingers, and slight trembling of the muscles. There was also numbness and deadness of the fingers of the left hand, with paresis. She could not pick up a penholder from the table. There was right wrist-drop, and she was completely unable to extend the fingers or wrist: she could not fully extend the fingers or wrist of left hand, nor keep the wrist extended against moderate pressure exerted on the back of it. The fingers were kept bent into the palm. The right thumb could not be properly opposed, and the action of the interossei of extending the two last phalangeal joints was lost; abduction of the fingers was partly retained. The right hand was constantly bathed in sweat, and the skin soft and sodden. The muscles of forearms were flaccid and soft, but not apparently wasted; they, as well as the muscles of the

* Buzzard, *Peripheral Neuritis;* Ross, Gowers, *loc. cit.*

legs, were very tender to touch. There was slightly diminished reaction to Faradaic, increased to Galvanic, current, more marked in right forearm than left. The feet and lower part of legs were in a constant state of perspiration, and the knee-jerks absent on each side. Her tongue was furred, appetite lost, and she complained of indigestion. She was ordered to leave off all alcohol, to have the muscles of the limbs well rubbed night and morning, and given pot. iod. with iron.

Six weeks later sensation had returned, there was increased power in hands, the wrist-drop much less, though the extensors were still weak ; the extensor action of interossei had not returned. Two months later still she had regained almost the normal use of her hands, and the hyperidrosis of hands and legs had gone.

This case was peculiar in that the paralysis affected the arms and not the legs, and the right arm much more than the left : it is interesting to note that vaso-motor paralysis appeared to take the place in the feet and legs of motor and sensory paralysis. The absence of knee-jerk, muscular tenderness, symmetrical tendency of the paralysis, and improvement in her condition on reforming her mode of life, appear all to point to alcoholic neuritis. The paralysis of interossei and thumb muscles was more severe than usual, as in a case recorded by Dr. Buzzard.

The next two cases come under the head of idiopathic neuritis. In the first there was a doubtful history of syphilis many years ago ; in the second there had been recent severe exposure to cold ; but in neither was there any evidence of the action of alcohol, lead, gout, or rheumatism.

July 16.—The first case is that of a woman aged 62, who had always been a teetotaller and healthy, and had had four healthy children. When a girl she lost the use of her left arm, which was swollen, bright scarlet, and very painful. She was told she had inflammation of the nerves, and recovered after having been freely bled. She now complained chiefly of intense and agonising burning and shooting pains in the arms, with loss of power in arms and hands. The illness began two months previously with tingling and numbness in fingers, beginning in right thumb; there was gradually increasing weakness, purple patches appeared over the fingers, and large blisters over the hands and arms. On examination there was double wrist-drop, with almost complete loss of power in the hands and forearms; there was slight power of flexion, but none of extension. She could not raise her arms above her head. The hands and fingers were swollen, the skin œdematous and covered with large bullæ, and hyperæsthetic; there appeared to be also deep muscular tenderness—the Faradaic current caused too much pain to be borne, but the muscles of the forearms and hands acted more readily than normal to weak Galvanic currents; the tendon-reflexes were increased. The muscles appeared wasted, especially those of the left arm and forearm, and showed occasional fibrillar tremblings. Paralysis of the small muscles of hands was also complete. The lungs were emphysematous—there was a faint mitral systolic murmur; the other organs were normal. She complained much of loss of sleep from the pain. She was ordered arsenic and quinine, and the arms wrapped in cotton-wool and kept at rest.

On September 10th the skin over hands and forearms was glossy, with an erythematous eruption, but no bullæ. There was some slight return of power, and the pains had ceased; the muscles were tender, but there was loss of sensation over the skin of hands and wrists. (See Fig. II.)

She was now recommended to rub the arms with ol. morrh., and was given a mixture containing iron and quinine, strychnine and iodide of potassium.

In October, a weak constant current was passed through the arms daily. She now gradually improved; the pains occurred at times, but became much less; the loss of sensation improved. She gradually regained power over the muscles. On November 19th, the skin, which had peeled completely over the forearms and hands, had returned to its normal appearance; sensation was normal. She could perform most of the muscular actions of fingers and arms, but there was still considerable weakness, especially on the right side; wasting was less apparent. The muscles of arms and forearms reacted only to strong Faradaic currents—muscles of left limb and hands requiring strongest; the muscles of right thumb and both little fingers reacted with a long latent period, and a slow sustained contraction, that seemed to pass as a slowly-moving wave over the muscle, resembling the contraction of an involuntary muscle-fibre. R. D. still obtained in right thumb and little finger, but nowhere else. A C C greater than K C C with ten Leclanché cells in these muscles.

She was now treated with stronger constant current passed through both arms, and individual Faradaic stimulation of special muscles. On February 14th, note was made that strength, range of movements, sensation, and electrical reactions were natural; so that the patient was ill eight months before recovery took place. The case illustrates well the extreme pain and severe lesions of skin that may occur: the former attack, apparently of neuritis, in her girlhood, is of interest.

In the following case there was paralysis of feet and hands, both motion and sensation; recovery taking place in three and half months from onset. The patient was a healthy woman, æt. 45, with a good family history. She had brought up a large family of healthy children, and had never suffered from rheumatism, gout, syphilis, or any other illness. She stated that she had an abscess in her breast one month previously; when that healed, the present illness began with tingling and feelings as of pins and needles in the calves, which four days afterwards also occurred in the hands. She had dull aching pains in the legs and numbness of the hands and feet. She now complained of total inability to walk, except a very short way with much difficulty, and of loss of power in the forearms and hands, with numbness and loss of sensation over legs and forearms, and of shortness of breath. She expressed herself as feeling that she had no feet when walking. She suffered no pain when at rest; and there had been neither lightning- nor girdle-pains, nor gastric crises. The tingling was passing off, and the loss of sensation becoming more marked. When she walked, she shuffled along with short steps, slowly and carefully, not taking her eyes off her feet, which she could not lift from the ground, being quite unable to walk upstairs. She could not walk in the dark without falling. She stood fairly steadily. She had lost all power of grasp, could not pick up anything from the table, nor fasten or unfasten the buttons of her jacket.

*On examination*, there was loss of sensation to pain and touch over forearms and legs. The distribution of the anæsthesia is indicated in Figure III.

There was double wrist-drop, deficient movement of fingers and loss of strength in forearms, and difficulty of raising hands to back of head. R.D. present in muscles of forearm and hand; former reacted, however, to very strong

Faradaic currents. Both feet were dropped; R.D. in muscles of both feet and legs. The quadriceps extensor acted only to very strong Faradaic currents. The muscles of calves and on front of legs were flaccid, flabby, and soft. In forearms these conditions were not so noticeable, except in muscles of thumbs and little fingers.

*Reflexes :* Bicipital and tricipital present; plantar, knee-jerk, absent. Front-tap contraction was obtained; abdominal reflexes present; no paralysis of muscles of trunk was observed. Skin normal. No swelling of feet. Organs examined and found normal. Micturition and defæcation normally performed.

Three weeks later, sensation was returning in feet, but not in hands, and there was some hyperæsthesia and pain in legs. The feet were still dropped, but less so, and she had gained in power in both hands and feet. Seven weeks later, she walked into my room very fairly, and had managed to walk a mile and a half to the Hospital without much discomfort. She could pick up and hold things; unbutton or button her jacket well. No wrist- or ankle-drop. She felt weak Faradaic current with brush everywhere, but least on palmar surface of fingers and soles of feet.

*Reflexes :* Superficial palmar exaggerated; knee-jerk present; no clonus or front-tap contraction ; no rigidity, no wasting of muscles apparent. The muscles reacted fairly well to Faradaic current, but to a weaker one in right than left; they reacted in forearms to a constant current of 10 cells Leclanché, in legs to one of 20 cells. K C C was greater than A C C, and A O C than K O C.

Treatment consisted merely in daily frictions of the limbs with oil, and iodide of potassium, with strychnine and iron and quinine, given internally.

The points of interest in this case are, first, the rapid and complete recovery from severe paralysis in about three months from onset; and, secondly, the absence of any apparent cause of the illness. There was not the slightest reason to doubt her statement, that she had always been extremely temperate as regards alcohol.

The following is a brief account of a case of neuritis affecting the forearm and hand of one side only, coming on entirely without pain, and without any loss of sensation to ordinary methods of examination. The patient was a strong healthy-looking labourer, æt. 66, who had had syphilis, but no other illness. He had been much exposed to wet lately, and complained of loss of power in the hand; he found it out from being unable to button his coat or to hold anything. The elbow was flexed, there was partial wrist-drop, and the hand lay to the ulnar side, with fingers partly flexed. There was some loss of power in movements of elbow, and he found a difficulty in raising his arm above his head. He could only slightly flex, and could not extend, fingers at all. Flexion and extension of wrist very imperfect. The actions of intrinsic muscles of hand were lost. Sensation—tested by a hair lightly drawn over the surface, and by gentle pricks with two needles—appeared normal, both as to touch and pain, over forearm and hand; but on careful examination with wire-brush and weak Faradaic current, sensation was deficient on left side as compared with right. There was tenderness, with apparent enlargement of trunks, of median and ulnar nerves. R.D. muscles of hand; loss of reaction to Faradaic current for muscles of forearm.

It is enough to say that this man recovered in six weeks under the above treatment. The case illustrates well the preponderance of motor over sensory paralysis that may exist. The tenderness and apparent swelling of affected nerve-trunks is interesting from their presence in a case of almost purely motor paralysis.

Now as to the localisation of the lesion in peripheral neuritis: from the presence of muscular atrophy, it must be situated either in the muscles themselves, in the nerves leading to them, or in the motor cells in the anterior horns. The presence of sensory disorders places the lesion in the nerves or in the grey matter of the cord. It has been argued that the lesion cannot be situated in the mixed nerves because, in some varieties, there is no affection of sensation throughout. I believe, however, that careful examination with delicate tests will in most cases detect some loss of sensation, though to rougher methods of examination this may have appeared normal; and it is well known that, in comparison with the motor disorder, a lesion of a mixed nerve may give rise to very trifling sensory disturbance. Secondly, Ross has pointed out that the lesion of the nerve frequently occurs below the point of origin of the sensory branch: for instance, the absence of sensory disturbance in lead-palsy is due to the fact that the musculo-spiral gives off its sensory fibres above the elbow as the radial nerve, and that lesion of the nerve occurs below the point of origin of the radial. Lastly, post-mortem examination in numerous cases has discovered a healthy spinal cord and changes in the nerves.

Acute anterior poliomyelitis presents clinically many points of resemblance to multiple neuritis, but is distinguished from it by the suddenness of its onset, and the far greater prominence of motor over sensory symptoms, while the sharp shooting pains and tenderness of nerve-trunks so often present in neuritis are absent. Atrophy of muscles is common to both. In some cases of multiple neuritis, where the motor disturbance is rapid and severe, and unaccompanied by sensory disorder, it is impossible to make the diagnosis at first.

The opinion seems to be gaining acceptance that under the name of infantile paralysis are included two distinct classes of cases: the one, cases of anterior poliomyelitis; the other, cases of multiple neuritis. The latter include all the cases in which recovery is almost complete, even after paralysis and atrophy have lasted a long time; in the cases due to poliomyelitis, there is no recovery after the disease has lasted a short time. Dr. Chapin, of New York, has recently reported * cases of infantile paralysis in which there was wrist- and ankle-drop, with the other characteristic symptoms of multiple neuritis; and also a very instructive case, in a child, where both the cord and the nerves were found diseased. He states that infantile paralysis due to anterior poliomyelitis generally occurs below the age of five, and nearly always before nine, years; and lays stress on the sudden onset, regressive character of paralysis, with absence of pain, as opposed to the ascending progressive palsy due to neuritis. Chronic anterior poliomyelitis is distinguished by the absence of sensory disturbance, and the order in which the muscles are involved (*i.e.* in the ascending form, small muscles of hand before extensors of fingers and forearm; in the descending, gluteal muscles and those supplied by obturator and anterior crural nerves before extensors of legs).

Paralysis of physiologically related muscles has also been regarded as pathognomonic of poliomyelitis in contrast to paralysis of muscles supplied by one nerve in

* *Med. Record*, Jan., 1887.

neuritis; but it is doubtful whether this rule can be regarded as absolute, as cases of multiple neuritis have lately been published where the first condition obtained.

Cases of spinal meningitis, with pressure on, and destruction of, nerve-roots, may resemble multiple neuritis very closely. Severe shooting pains along the course of the nerves, loss of sensation, and paralysis with atrophy of muscles, are common to both; but in meningitis of this severity there is interference with micturition and defæcation, the loss of sensation is very irregularly distributed, and there are generally pains and stiffness in the back, hyperæsthesia, and girdle-pains.

From Landry's paralysis, acute cases of multiple neuritis will be distinguished by the absence of sensory disorders, muscular wasting, and the reaction of degeneration in the former.

In the diagnosis, it is important to remember in all cases that in patients suffering from multiple neuritis, any interference with the functions of the bladder or rectum is of extremely rare occurrence.

To tabes dorsalis, cases of widely distributed paralysis from multiple neuritis have considerable resemblance; and this derives especial interest from the fact that Pitres and Vaillard have shown that peripheral neuritis frequently occurs in this disease. They say that the distribution of the neuritis in cases of tabes varies widely, and bears no relation to the extent of the lesion in the cord; that it probably plays no part in the production of the specific symptoms of tabes—*e.g.* lightning-pains, absence of knee-jerk, inco-ordination; but that, on the other hand, the areas of cutaneous analgesia and anæsthesia, cutaneous trophic changes such as perforating ulcer, motor paralyses with or without muscular atrophy, spontaneous fractures and arthropathies, and perhaps also visceral crises—symptoms all of which frequently occur in the victims of tabes—are due to peripheral neuritis.

Ross* lays stress on the gait in the diagnosis, which has been compared to that of a high-stepping horse, or of a dancing-master. The following is his explanation of it: Suppose that one foot has just been moved forwards and planted on the ground in front of the other; the body is now inclined to that side, and at the same time the heel of the foot in the rear is raised and the limb is ready to move forwards; the anterior muscles of this leg now contract, so that the toes are raised and clear the ground, and the leg now swings forward by its own weight like a pendulum, and not by any further muscular action. But in multiple neuritis, the anterior muscles of the leg are paralysed and unable to raise the toes; so that when the foot is raised the toes simply drop lower, and the heel must be unusually elevated to enable the foot to be moved forwards, thus exposing more of the sole to an observer from behind than is usual, and the necessary elevation of the toes is obtained by a further contraction of the muscles that flex the knee on the thigh and the thigh on the pelvis.

Of course, the general symptoms of the two diseases, such as eye-symptoms, course of the disease, &c., are widely different.

With regard to prognosis while the paralysis is still spreading, there is necessarily some anxiety lest it should involve the muscles of respiration; but Buzzard says that, even when this happens, most cases recover. The prognosis necessarily varies according to the cause of the disease in each individual case: it is best in diphtheritic cases, where death rarely occurs directly from paralysis;

* *Loc. cit.*

in alcoholic cases, according to most English observers, recovery generally takes place, but the patient, on regaining his liberty, almost invariably returns to his previous intemperate habits, and a relapse follows. According to French physicians, the prognosis is very grave in this form of the disease. It must be borne in mind that the patient is often at the lowest ebb of nutrition when he comes under treatment for paralysis.

In the forms of gouty or rheumatic, or in those classed above as idiopathic neuritis, recovery generally takes place after a longer or shorter period. In short, in all varieties of peripheral neuritis, the tendency is probably to recovery, provided that the cause of the disease can be removed. As to the time taken for recovery, that varies within wide limits, and of course depends in part on the extent to which degeneration in the muscles and nerves has taken place. The electrical reactions afford exact information, other things being equal, with regard to the interval that must elapse before restoration of function.

The treatment should consist of rest in the early stages, with measures for the relief of pain if severe. After the first stage, a weak constant current should be passed through the nerves in both directions. Mercurial inunction is useful in recent syphilitic cases. Iodide of potassium, in fairly large doses, should be given with tonics. Alcoholic stimulants are not generally desirable. When the paralysis has ceased to spread, a constant current, slowly made and broken, and just strong enough to cause contraction, should be passed daily through the affected muscles; daily frictions of the affected limbs are also of advantage. Later still, when recovery begins, Dr. Allen Starr thinks that the restoration of the normal passage of impulses through the nerves is hastened by passing an interrupted current through them. The Faradaic current may be useful in the latest stages of slowly improving cases, when the muscles respond to it, but are still weak and wanting in tone; but it is doubtful whether it materially hastens recovery, and it is useless or injurious when applied to the muscles earlier. In cases where the anæsthesia is great, advantage is sometimes derived from daily stimulation of the skin of the affected parts by the Faradaic brush, as recommended by Duchenne. Arsenic and strychnine are very useful after the first stages. I think a mixture containing iodide of potassium and strychnine, with the citrate of iron and quinine, gives the best results.

## Figures and Tables

**Fig 1 Fig 2 f1:**
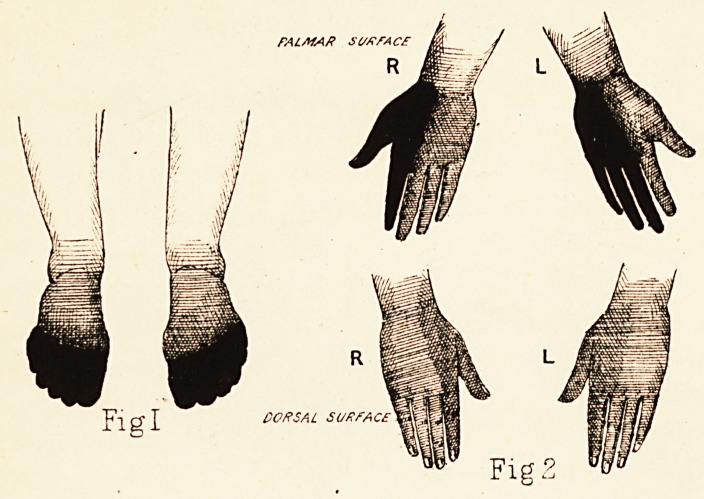


**Fig 3 f2:**